# The Connecticut Veterinary Medical Society

**Published:** 1896-07

**Authors:** H. W. Eliot

**Affiliations:** Secretary


Secretary.

### THE CONNECTICUT VETERINARY MEDICAL SOCIETY.

THE annual meeting of this Society was held at the office of Dr. Thomas Bland, in Waterbury, on the evening of June 20, 1896. Drs. Whitney, Kelley, and Isaacs, of New Haven; Lyman, of Hartford; Storrs, of Willimantic; Bland, of Waterbury; and Eliot, of Ansonia, answered to roll-call. Drs. Keeley and McKenna were present as guests of the Association. A communication from Dr. Ross, of New Haven, stating that he could not be present, and wishing us an interesting and instructive meeting, was read.

The Board of Censors reported favorably on the applications for membership of Dr. R. P. Lyman, of Hartford, and Dr. R. D. Martin, of Bridgeport, and they were elected. The application of Dr. Ingram, of Hartford, was not acted upon.

Applications for membership were received from Dr. P. S. Keeley, a graduate of the New York College of Veterinary Surgeons, and Dr. E. P. McKenna, a graduate of the Veterinary Department of Harvard University.

The election of officers for the ensuing year was as follows: President, Dr. Harrison Whitney, New Haven; First Vice-President, Dr. E. R. Storrs, Willimantic; Second Vice-President, Dr. R. P. Lyman, Hartford; Secretary, Dr. H. W. Eliot, Ansonia; Treasurer, Dr. J. H. Kelley, New Haven; Board of Censors, Drs. J. E. Gardner, Hartford; R. P. Lyman, Hartford; Thomas Bland, Waterbury; M. Isaacs, New Haven; R. D. Martin, Bridgeport.

Dr. Bland then gave a report of an operation that he performed on the mare Vandetta, owned by F. A. Coe, of Middlefield, Conn. The mare had stood in her stall all winter. The first time she was taken out for exercise it was noticed that she wheezed a little, and kept growing worse. A veterinarian was called and diagnosed the trouble as acute laryngitis, and prescribed appropriate remedies. The breathing kept growing worse till it was necessary to insert a tracheotomy tube, which at once gave relief. Temperature and pulse always remained nearly normal. Dr. Bland, on an internal examination of the throat, discovered a large growth at the base of the tongue, extending to the epiglottis. This growth, pressing backward on the epiglottis, closed the opening of the larynx, which prevented the horse from breathing. Instruments were at once made for an operation on the growth, which proved to be a mucous cyst. As soon as this was opened and the contents evacuated the horse was once more able to breathe in a natural way. The horse died in about two weeks after from septic pneumonia. Dr. Bland made a post-mortem and secured the larynx and part of the tongue, which he showed to the members present. The walls of the cyst and the opening into it could still be seen.

After the meeting adjourned a banquet was served at Batchaler's.

The next meeting will be held at Hartford, Conn.

H. W. Eliot, V.S.,
Secretary.

